# Medial meniscal ramp lesions in ACL-injured elite athletes are strongly associated with medial collateral ligament injuries and medial tibial bone bruising on MRI

**DOI:** 10.1007/s00167-021-06671-z

**Published:** 2021-08-03

**Authors:** Lukas Willinger, Ganesh Balendra, Vishal Pai, Justin Lee, Adam Mitchell, Mary Jones, Andy Williams

**Affiliations:** 1 Fortius Clinic, 17 Fitzhardinge St, London, W1H 6EQ UK; 2 Department of Trauma Surgery, Technical University of Munich, Ismaninger Straße 22, 81675 Munich, Germany

**Keywords:** Ramp lesion, Menisco-capsular separation, Magnetic resonance imaging, Arthroscopy, Risk factors, Association

## Abstract

**Purpose:**

Medial menisco-capsular separations (ramp lesions) are typically found in association with anterior cruciate ligament (ACL) deficiency. They are frequently missed preoperatively due to low MRI sensitivity. The purpose of this article was to describe demographic and anatomical risk factors for ramp lesions, and to identify concomitant lesions and define their characteristics to improve diagnosis of ramp lesions on MRI.

**Methods:**

Patients who underwent anterior cruciate ligament (ACL) reconstruction between September 2015 and April 2019 were included in this study. The presence/absence of ramp lesions was recorded in preoperative MRIs and at surgery. Patients’ characteristics and clinical findings, concomitant injuries on MRI and the posterior tibial slope were evaluated.

**Results:**

One hundred patients (80 male, 20 female) with a mean age of 22.3 ± 4.9 years met the inclusion criteria. The incidence of ramp lesions diagnosed at surgery was 16%. Ramp lesions were strongly associated with injuries to the deep MCL (dMCL, *p* < 0.01), the superficial medial collateral ligament (sMCL, *p* < 0.01), and a small medial–lateral tibial slope asymmetry (*p* < 0.05). There was also good correlation between ramp lesions and bone oedema in the posterior medial tibia plateau (MTP, *p* < 0.05) and medial femoral condyle (MFC, *p* < 0.05). A dMCL injury, a smaller differential medial–lateral tibial slope than usual, and the identification of a ramp lesion on MRI increases the likelihood of finding a ramp lesion at surgery. MRI sensitivity was 62.5% and the specificity was 84.5%.

**Conclusion:**

The presence on MRI of sMCL and/or dMCL lesions, bone oedema in the posterior MTP and MFC, and a smaller differential medial–lateral tibial slope than usual are highly associated with ramp lesions visible on MRI. Additionally, a dMCL injury, a flatter lateral tibial slope than usual, and the identification of a ramp lesion on MRI increases the likelihood of finding a ramp lesion at surgery. Knowledge of the risk factors and secondary injury signs associated with ramp lesions facilitate the diagnosis of a ramp lesion preoperatively and should raise surgeons’ suspicion of this important lesion.

**Level of evidence:**

Diagnostic study, Level III.

## Introduction

“Ramp lesions” were first described by Strobel [47] in 1988 to define a menisco-capsular separation of the posterior horn of the medical meniscus (PHMM) form the posteromedial capsule (PMC) and they are most commonly found in association with anterior cruciate ligament (ACL) ruptures [3, 6, 11, 27, 31, 32, 41, 45]. The PHMM is firmly attached to the PMC [12, 52] and it acts as a secondary knee stabiliser to resist anterior translation of the medial tibia and thereby external tibial rotation [1, 37, 46]. In the event of an acute ACL injury, the forceful forward displacement of the tibia and subsequent stress on the posteromedial capsule and PHMM can result in posteromedial menisco-capsular injury—a ramp lesion. These occur in 9–34% of patients with ACL tears at the time of ACL rupture [3, 6, 11, 27, 31, 32, 41, 45].

The word ‘ramp’ refers to the appearance of the synovium/PMC that is seen to sweep proximal and anterior, like a ramp, to the posterior margin of the PHMM when the posteromedial recess is viewed in the flexed knee. In the extended knee, the posterior capsule tightens, and pulled proximally and thereby obliterating the posteromedial recess and making the ‘ramp’ disappear [46]. It is in the extended position that magnetic resonance imaging (MRI) of the knee is usually undertaken, thus compromising detection of ramp lesions since there is no ‘ramp’. This phenomenon may account for the low sensitivity of MRI in identifying ramp lesions, which means ramp lesions are frequently not diagnosed preoperatively [3, 11]. The intraoperative detection through a systematic arthroscopic exploration, including a direct posteromedial visualization through the intercondylar notch or a direct posteromedial portal, currently remains the gold standard to detect a ramp lesion and can be technically difficult.

Knowledge of the associated factors and secondary injury signs associated with ramp lesions will facilitate the diagnosis of a ramp lesion on MRI. Increased preoperative suspicion of a ramp lesion would be invaluable to the surgeon at time of arthroscopy so that lesions would not be overlooked at surgery. The purpose of this study was to describe demographic and anatomical associated factors for ramp lesions in elite athletes, to identify associated lesions on MRI and define their characteristics. The MRI findings were correlated with operative findings. It was hypothesized that a steeper medial slope is a risk factor for ramp lesions and that bone oedema at the posterior medial tibial plateau (MTP), and medial collateral ligament (MCL) injuries are associated with these injuries, given that it is logical that the injury mechanics causing the ramp lesion are those occurring with AMRI.

## Materials and methods

This study was conducted according to the UK National Health Research Authority guidance and ethically approved by the institution (Fortius Clinic, London, UK) involved. This retrospective cohort study comprised a consecutive series of professional athletes who underwent ACL reconstruction between 2015 and 2019. This group was specifically chosen as they have MRI scans and surgery consistently soon after injury and, therefore, the MRI and surgery would occur with little delay from injury allowing best contemporaneous correlation of clinical, arthroscopic and MRI findings. Patients eligible for inclusion in the study were identified by a review of medical records, and demographic information, injury data, time from injury to MRI and surgery, as well as intraoperative findings were recorded. Patients were excluded if there was any history of previous ipsilateral knee injury or surgery, and any concurrent laxity or surgery of knee ligaments other than the ACL. To allow for high levels of accuracy in evaluation of damage to peripheral structures, only patients with an MRI scan taken within 3 weeks of the ACL injury, and that met the minimum imaging criteria of (1) field strength of 1.5 Tesla or above, (2) three-plane (sagittal, axial and coronal) imaging using water sensitive fat-suppressed sequences (STIR, fat-suppressed proton density or T2-weighted) and (3) slice thickness of 3 mm or less were included.

### Radiological assessment

Preoperative MRI examinations were acquired from multiple centres. Due to the consequent variation in scanning protocols the sequences used varied. They often included T1-weighted imaging. However, for the purpose of the study, and to maintain consistency, only the fluid sensitive sequences were used for image analysis.

**Fig. 1:**
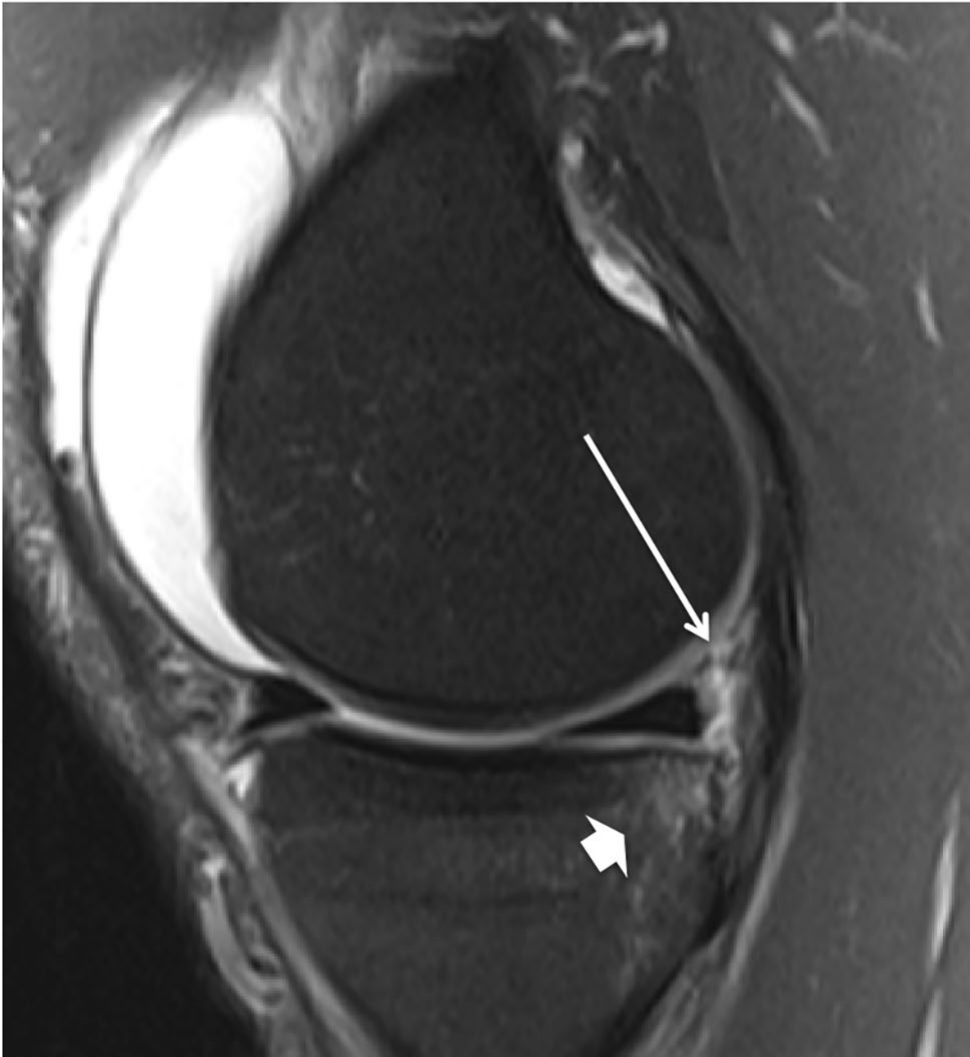
Sagittal fat-suppressed proton density weighted MRI shows a separation of the posteromedial capsule and the posterior horn of the medial meniscus (ramp lesion, long →) and bone oedema at the posterior medial tibial plateau (thick →)

Two radiologists specialized in musculoskeletal imaging with 20 and 25 years of experience, respectively, independently analysed all MRI images. Occurrence of a ramp lesion was recorded if there was fluid signal separating the PMC and the PHMM (Fig. 1). In addition, the presence or absence, and location of bone oedema in the medial compartment was recorded. Bone oedema was defined as increased signal intensity on the fat-suppressed water sensitive images within the bone. Injuries to the superficial and deep medial collateral ligament (sMCL, and dMCL), posterior oblique ligament (POL), lateral collateral ligament (LCL), anterolateral complex including the anterolateral ligament (ALL) and Kaplan fibres (KF), and the menisci were also recorded.

The technique used in this study for measurement of medial and lateral posterior tibial slope has been previously published and validated by other authors [22, 28, 33]. The posterior tibial slope was defined as the difference between the sagittal tibial joint surface orientation and a perpendicular line to the proximal anatomical tibial axis. A larger positive value indicates a steeper posterior tibial slope. Medial–lateral slope differential was calculated by subtracting the medial tibial slope from the lateral medial slope.

### Ligament laxity assessment

All patients were routinely examined under anaesthesia (EUA) at the beginning of surgery by the senior author who is a specialist sports knee surgeon with over 25 years’ experience. This included anterior and posterior drawer, Lachman, pivot-shift test, dial test, valgus and varus stress tests. The relevant stress tests were categorized according to International Knee Documentation Committee form (grade I: 3–5 mm, grade II: 5–10 mm, grade III: > 10 mm) as laxity differences compared to the healthy contralateral knee [20]. The pivot-shift test was graded as 0 (equal), 1 (glide), 2 (clunk), or 3 (gross).

### Arthroscopic assessment

**Fig. 2:**
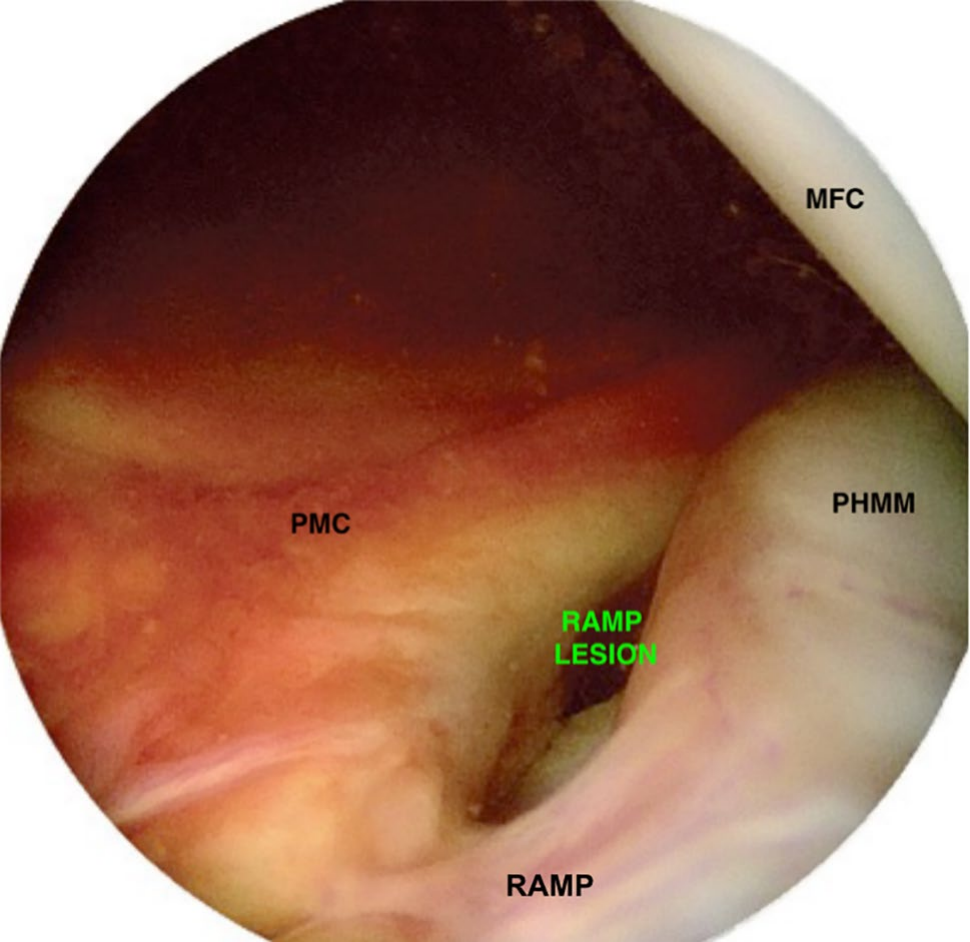
Arthroscopic trans-notch view through the anterolateral portal with a 30° camera reveals a menisco-capsular separation (ramp lesion) of a right knee. *PHMM* posterior horn of the medial meniscus, *PMC* posterior medial capsule, *MFC* medial femoral condyle

Standard anteromedial and anterolateral portals were made for ACL reconstruction surgery and a routine diagnostic assessment was made of the suprapatellar pouch, patellofemoral joint, lateral gutter including popliteus tendon and hiatus, medial gutter, medial compartment, intercondylar notch and lateral compartment. The posteromedial compartment and ramp were assessed by advancing a 30° arthroscope over the anterior surface of the ACL stump into the posteromedial recess through the intercondylar notch. To aid this the knee is held in 30° flexion with a varus stress applied (Gillquist maneuver [15, 30]). Some authors recommend inserting the arthroscope via an accessory posteromedial portal to visualise the ramp region [51]. However, this is not the senior author’s routine as ramp lesions are revealed as knee flexion was increased (Fig. 2). Occasionally a posteromedial portal is used to insert a probe.

### Statistical analysis

Data were analysed using SPSS statistics software version 23.0 (IBM, New York, USA). Normal distribution was confirmed by the Shapiro–Wilk test and continuous variables were expressed as mean ± standard deviation. Chi-squared test or Fisher’s exact test was used to analyse for any association between ramp lesions and demographic variables, EUA and other MRI findings. Binomial logistic regression analysis was performed to evaluate the associated factors for the presence of ramp lesions. The included six predictive factors were chosen by background knowledge and were medial and lateral tibial slope, and the presence of sMCL and dMCL injury, MRI ramp injury and bone oedema at the medial tibial plateau (MPT). Cohen’s kappa value has been calculated for inter-rater agreement to detect ramp lesions and inter-rater correlation coefficient (ICC) was calculated for inter-rater reliability for measuring medial and lateral tibial slope A post hoc power analysis revealed an actual power of 82% for finding differences between two independent proportions (p1 = 0.25, p2 = 0.62) with a group allocation of 84:16 subjects (intact vs. ramp lesion) (G*Power 3.1). Statistical significance was set at a *p* value of < 0.05.

## Results

One hundred and fifty-three patients underwent ACL reconstruction during the study period and of these 100 (80 male and 20 female) with a mean age of 22.3 ± 4.9 years met the inclusion criteria. The 53 patients were excluded due to concomitant medial and/or lateral abnormal knee laxity or for failing to meet the minimum imaging criteria. There were 81 non-contact and 19 contact injuries in 53 right and 47 left knees. All patients were professional athletes and included 60 soccer players, 26 rugby players and 14 players from other sports. The median time between injury and MRI was 2 days (0–21) and 13 days (3–100) between injury and surgery. Cohen’s kappa analysis showed excellent agreement between the two readers for the assessment of ramp lesions (1.00, *p* < 0.001). The ICC value for reliability of MRI measurements was 0.892 (95% CI 0.43–0.925, *p* < 0.01) for medial tibial slope and 0.977 (95% CI 0.966–0.985, *p* < 0.01) for the lateral tibial slope, indicating excellent agreement.

### Incidence of ramp lesions

The incidence of ramp lesions diagnosed on preoperative MRI was 23% compared to a ‘diagnosis at surgery’ rate of 16%. Of these, 15 were repaired and only one was deemed stable and left alone. Hence, the MRI sensitivity to identify a ramp lesion was 62.5%, the specificity was 84.5%, the positive predictive value was 43.5% and negative predictive value was 92.2%. There was no difference in the timing of the MRI or surgery between the groups with and without ramp lesions.

### Association with ramp lesions

Results of knee laxity EUA and preoperative MRI for the presence of intraoperative ramp lesions are summarized in Tables 1 and 2. Patients’ gender, age and injury mechanisms did not correlate with the presence or absence of ramp lesions at surgery.

**Table 1:** Patients’ EUA results in relation to the presence of intraoperative meniscal ramp lesions

	Meniscus ramp		*p* value
	Intact	Injured	
Anterior drawer test			
Grade I	69 (82.1%)	14 (87.5%)	n.s
Grade II	15 (17.9%)	2 (12.5%)	
Lachman test			
Grade I	2 (2.4%)	0 (0%)	n.s
Grade II	31 (36.9%)	4 (25.0%)	
Grade III	51 (60.7%)	12 (75.0%)	
Pivot shift test*			
Grade 1	25 (30.9%)	4 (26.7%)	n.s
Grade 2	47 (58.0%)	7 (46.6%)	
Grade 3	9 (11.1%)	4 (26.7%)	

Data is given as numbers (%)

*Data are only available for 96 patients

**Table 2:** Concomitant injuries on MRI (i.e. the presence of oedema) in relation to the presence or absence of intraoperative meniscal ramp lesions

	Meniscus ramp		*p* value
	Intact	Injured	
Superficial MCL			
Intact	39 (46.4%)	1 (6.3%)	***p* = 0.002**
Injured	45 (53.6%)	15 (93.8%)	
Deep MCL			
Intact	63 (75.0%)	6 (37.5%)	***p* = 0.006**
Injured	21 (25.0%)	10 (62.5%)	
POL			
Intact	75 (89.3%)	14 (87.5%)	n.s
Injured	9 (10.7%)	2 (12.5%)	
LCL			
Intact	78 (92.9%)	15 (93.8%)	n.s
Injured	6 (7.1%)	1 (6.3%)	
Kaplan fibres			
Intact	36 (42.9%)	3 (18.8%)	n.s
Injured	48 (57.1%)	13 (81.3%)	
Anterolateral ligament			
Intact	67 (79.8%)	12 (75.0%)	n.s
Injured	17 (20.2%)	4 (25.0%)	
Medial meniscus			
Intact	56 (66.7%)	10 (62.5%)	n.s
Injured	28 (33.3%)	6 (37.5%)	
Lateral meniscus			
Intact	37 (44.0%)	8 (50.0%)	n.s
Injured	47 (56.0%)	8 (50.0%)	
MTP bone oedema			
Absent	40 (47.6%)	2 (12.5%)	***p* = 0.012**
Present	44 (52.4%)	14 (87.5%)	
MFC bone oedema			
Absent	60 (71.4%)	7 (43.8%)	***p* = 0.043**
Present	24 (28.6%)	9 (56.3%)	
Medial tibial slope (°)	3.3 ± 2.3	3.7 ± 2.3	n.s
Lateral tibial slope (°)	7.0 ± 3.7	5.4 ± 2.9	n.s
Tibial slope asymmetry (°)	3.8 ± 3.9	1.7 ± 2.8	***p* = 0.044**

Data is given as numbers (%)

*MCL* medial collateral ligament, *POL* posterior oblique ligament, *LCL* lateral collateral ligament, *MTP* medial tibial plateau, *MFC* medial femoral condyle

There was no statistically significant correlation between the presence of ramp lesions and the grade of anterior drawer test, Lachman test and pivot-shift test (Table 1). However, 12 of 16 patients (75%) with a ramp lesion had a grade III Lachman test.

93.7% of patients with ramp lesions exhibited concomitant sMCL injury (the presence of oedema in the sMCL—but with normal clinical laxity) on MRI. Ramp lesions are strongly associated with injuries to the sMCL (*p* = 0.002, OR 13.000, 95% CI 1.642–102.936), and the dMCL (*p* = 0.006, OR 5.000, 95% CI 1.621–15.419). There was also a strong correlation between ramp lesions and bone oedema in the posterior MTP (*p* = 0.012, OR 6.364, 95% CI 1.361–29.750) and MFC (*p* = 0.043, OR 3.214, 95% CI 1.075–9.611). In addition, differential medial–lateral posterior tibial slope was significantly smaller in patients with a ramp lesion compared to patients with an intact meniscus ramp (*p* < 0.05). Ramp lesions were not associated with oedema in the POL, LCL, Kaplan fibres, or ALL, nor medial or lateral meniscus lesions (Table 2).

### Associated factors for ramp lesions

Associated factors associated with the ramp lesions seen at arthroscopy were identified with logistic regression analysis with backward elimination (Table 3). The presence of a MRI ramp lesion, dMCL injury and a flatter lateral tibial slope was statistically significant associated factors for the presence of ramp lesion at surgery. Finding a ramp lesion intraoperatively is more likely if it is detected on MRI (OR 14.87). The risk of having a ramp lesion was significantly raised by a simultaneous dMCL (OR 6.45). A flatter lateral tibial slope increased the risk of having a ramp lesion with each degree by 1.3 times.

**Table 3:** Logistic regression analysis shows the association between the presence of an intraoperative ramp lesion and clinical and radiological factors

Factor	Odds ratio	95% CI	*B* ± SE	*p* value
Lateral tibial slope	0.761	0.606–0.955	− 0.274 ± 0.116	***p* = 0.019**
dMCL injury	6.449	1.459–28.504	1.864 ± 0.758	***p* = 0.014**
MRI ramp lesion	14.870	2.914–75.872	2.699 ± 0.321	***p* = 0.001**
Constant value			− 4.041 ± 1.339	***p* = 0.018**

Odds ratio describes the risk of exhibiting a ramp lesion. Nagelkerke *R*^2^ = 0.48

*CI* confidence interval, *dMCL* deep medial collateral ligament

## Discussion

The main findings of this study were that the presence on MRI of posterior MTP and MFC oedema, sMCL and dMCL lesions and a smaller tibial slope asymmetry (1.7° vs. 3.8°) are highly associated with ramp lesions. Additionally, with binomial logistic regression analysis, a dMCL injury, a flatter lateral tibial slope and the identification of a ramp lesion on MRI significantly increased the likelihood of finding a ramp lesion at surgery.

The medial meniscus, with its firm attachment to the tibia via the menisco-tibial ligament, and specifically the posterior horn, is a secondary restraint to anterior tibial translation and external rotation of the knee. Its function becomes even more important in ACL-deficient knees [1, 2, 26, 35]. Biomechanical studies have demonstrated that ramp lesions in addition to ACL deficiency, increases anteroposterior instability and external rotational instability [1, 46]. AMRI has also been found in clinical studies [6, 32, 49]. There is growing scientific evidence and consensus among knee surgeons that ramp lesions should be sought, identified and repaired [7, 38, 40, 50]. Repair of ramp lesions is safe [21] and restores normal knee kinematics when combined with ACL reconstruction in in-vitro studies [46]. Therefore, it is vital to detect ramp lesions, both on MRI and at surgery, and repair them at the time of ACL reconstruction or risk ongoing pain, instability and ACL graft failure due to overload. Traditionally ramp lesions were not detected as they cannot easily be identified when viewing the posterior medial meniscus with the arthroscope placed anteriorly in the medial compartment. The diagnostic requires to view the ramp region with the arthroscope in the posteromedial recess using the intercondylar approach or an additional posteromedial portal. As recommended, this inspection must be done routinely during ACL reconstruction procedures. MRI identification of a ramp lesion and its associated factors will alert a surgeon to focus to the posteromedial area.

As an identified ramp lesion on MRI increased the chance of finding it at surgery by 13.6 times it emphasises the importance of this knowledge. This study found that MRI had a moderate sensitivity (62.5%) but high specificity (84.5%), and a high negative predictive value (92.2%) in detecting ramp lesions. The published rates for MRI sensitivity in identifying ramp lesions are 48–90% [3, 11, 19, 27, 29, 49, 56] with 3 T MRI possibly superior to 1.5 T MRI scans [19]. Arner et al. [3] compared results of three MRI readers with arthroscopic findings. Their MRI sensitivity varied between 53.9 and 84.6%. This is similar to the findings of the present study, which showed a much higher inter-rater agreement, highlighting the benefit of having experienced MSK radiologists and of applying specific MRI criteria for ramp lesions. MRI specificity was reported to be over 90% in several studies [3, 19, 27], which is similar to the findings in this study of 84.5%. Overall these results indicate that preoperative MRI is not accurate enough to detect all ramp lesions but is an excellent modality to exclude the presence of ramp lesions. Therefore, routine inspection of the ramp region in the posteromedial recess during arthroscopy in knees with ACL injury is advocated.

The incidence of ramp lesions in ACL-injured knees at surgery in a professional athlete’s population in this study is 16%. This is consistent with the published literature in which the incidence is reported to range from 9 to 34% [3, 6, 11, 19, 27, 29, 31, 41, 56]. The difference of ramp lesions’ prevalence between contact and non-contact injuries was not significant in the present study, but has been described previously [41]. It is important to mention that although good views were obtained at surgery, it is possible that simply viewing the ‘ramp’ region with a 30° arthroscope passed into the posteromedial recess via the intercondylar notch and not using a posteromedial portal that the some ramp lesions could have been missed and, therefore, the incidence is under-reported.

In the present study, the presence of ramp lesions is strongly associated with concurrent MRI injury to the sMCL and especially the dMCL. Interestingly, ramp lesions were not associated with damaged to the posteromedial capsule, what could have been expected due to the close anatomical relation [8]. This finding links injuries to the posterior menisco-capsular junction of the medial meniscus with injuries to the medial collateral ligaments [23]. One of the roles of the MCL is resistance to anterior translation of the medial tibial plateau/external rotation for which the dMCL is the primary restraint between 0° and 60° knee flexion [4, 39, 53, 54] as well as valgus, for which the sMCL is the primary restraint. The association of concomitant injury to the dMCL and sMCL and ramp lesions is suggestive of a specific injury mechanism causing the ramp lesion as well as injury to the dMCL and sMCL. To injure these structures together logically there must be significant anterior translation and/or external rotatory subluxation of the medial tibial plateau during the event of an ACL rupture, since these loads are resisted by the posterior medial meniscus, dMCL and sMCL. This might cause the medial femoral condyle to move posteriorly and ride over the medial meniscus thereby stretching the PMC in the posteromedial recess to the point of failure [16]. In fact, 15 of 16 patients (93.7%) with ramp lesions had a sprain of the sMCL and 62.5% a lesion of the dMCL, which is firmly bound to the mid-portion of the medial meniscus. In contrast, the POL was intact in 87.5% patients, further reinforcing the idea that this injury is due to an anterior translation of the medial tibia /external rotation injury mechanism as the POL resists internal rotation in the knee close to full extension. Furthermore, bone oedema at the posterior MTP and the MFC was also correlated with ramp lesions, and 87.5% of cases with proven ramp lesions had MTP bone oedema, which is in keeping with the same mechanism. This finding is in agreement with previous studies that reported posteromedial tibial bone oedema as an important secondary MRI finding in conjunction with ramp lesions [5, 11, 27, 29]. DePhillipo et al. [11] found MTP bone bruises in 72% of their patients diagnosed with ramp lesions. In contrast, Hatayama et al. only found bone contusions in 38% of their patients with ramp lesions and the incidence of bone contusion did not differ from patients with an intact medial meniscus [19]. The higher incidence in the present study may reflect the patient population: perhaps injuries in professional athletes are more severe.

The present study also showed that a smaller posterior tibial slope asymmetry was associated with the ramp lesion and, from the logistic regression analysis, that a steeper posterior lateral tibial slope decreased the risk for them. In contrast, increased lateral tibial slope is associated with ACL rupture [10, 14, 18]. The authors of this present study believe that a higher lateral tibial slope predisposes to the lateral femoral condyle subluxing off the posterior lateral tibia causing ACL rupture and the classic lateral compartment distal femoral and posterior tibial bone bruises. In such cases the abnormal motion is predominantly in the lateral compartment with the centre of axial rotation on the medial tibial plateau leaving the ramp intact as the MFC does not move posteriorly. Conversely in knees with less lateral posterior slope laterally, and thus less differential medial/lateral posterior slope, with anterior tibial displacement the femur will have less tendency to move posteriorly just in the lateral component and will also do so medially hence loading the PHMM and PMC. Our results are similar to the findings of Kim et al. [27] who associated ramp lesions with a steeper medial and flatter lateral slope. Also Song et al. found a higher incidence of ramp lesions in patients with an increased medial slope [44].

With regard to clinical examination, Bollen described a correlation of ramp lesions with anteromedial rotatory subluxation [6]. The author noticed an increased anterior movement of the medial tibial plateau when the foot was externally rotated in 90° knee flexion [6] (i.e. Slocum test [42]). Ramp lesions have also been associated with a higher side-to-side difference of anterior translation examined with a KT-2000 [49] and a higher incidence of grade III pivot-shift [32]. This study could not identify a statistically significant association between the presence of ramp lesions and a higher grade of Lachman test, anterior drawer test or pivot-shift test. However, 12 of the 16 knees with a ramp lesion had grade 3 Lachman tests and the other 4 were grade 2. Failure to reach statistical significance could, therefore, be a type 2 error as 20% of studies suffer from failure to show a difference statistically when one actually exists [13]. Unfortunately, the Slocum test for anteromedial rotatory instability was not routinely performed during the present study.

This study has several limitations. All patients were professional athletes and, therefore, do not represent a typical patient cohort compared to most clinical practices, and their injury patterns and rates of ramp lesion may not be the same as in other patient groups. These patients were, however, chosen to ensure the best quality of MRI imaging, and scans sufficiently soon after injury to document the full extent of injury to intra-articular structures and the soft tissue envelope as they have more immediate access to MRI examination. Since these patients tend to have early surgery direct correlation of MRI findings with arthroscopic diagnosis is possible. The situation can change as delay might allow time for healing of ramp lesions. This does bring into to question the possibility that early surgery increases diagnosis and possible over treatment of ramp lesions that might otherwise heal. Furthermore, the size and category of ramp lesions (stable or unstable) was not further classified and analysed.

In addition, MRIs were, however, acquired in various institutions with different protocols that could affect scan quality, but minimum imaging requirements were applied for inclusion in the study design to allow for reliable analysis, which was reflected by high inter-rater agreement. An obvious issue is that whilst abnormal (high) signal represents injury to tissue, the integrity or otherwise of that tissue cannot be certain. Furthermore, spread of oedema / hematoma might, by MRI criteria, imply injury to soft tissues that are, in fact, intact. Again, this risk is mitigated by the scans mainly being undertaken 2 or 3 days from injury.

The results from the present study emphasize the relation between ramp lesions and damage to the medial soft tissue envelope, hence, indicating a likely external rotation injury mechanism in some ACL ruptures. This should raise awareness of possible AMRI in these patients which need to be carefully assessed through clinical examination and addressed in the operating room.

## Conclusion

In cases of acute ACL rupture, on MRI the presence of bone oedema in the MFC and posterior MTP, sMCL and dMCL lesions, and a smaller differential medial–lateral tibial slope are highly associated with ramp lesions. Additionally, a dMCL injury, a flatter lateral tibial slope, and the identification of a ramp lesion on MRI increase the likelihood of finding a ramp lesion at surgery according to logistic regression analysis. Preoperative MRI has only moderate sensitivity, but high specificity, and a high negative predictive value for detection of ramp lesions.
